# Cholesterol overload in the liver aggravates oxidative stress-mediated DNA damage and accelerates hepatocarcinogenesis

**DOI:** 10.18632/oncotarget.22024

**Published:** 2017-10-24

**Authors:** Cristina Enríquez-Cortina, Oscar Bello-Monroy, Patricia Rosales-Cruz, Verónica Souza, Roxana U. Miranda, Rafael Toledo-Pérez, Armando Luna-López, Arturo Simoni-Nieves, Rogelio Hernández-Pando, María Concepción Gutiérrez-Ruiz, Diego F. Calvisi, Jens U. Marquardt, Leticia Bucio, Luis Enrique Gomez-Quiroz

**Affiliations:** ^1^ Posgrado en Biología Experimental, DCBS, Universidad Autónoma Metropolitana Iztapalapa, México City, México; ^2^ Laboratorio de Fisiología Celular, Departamento de Ciencias de la Salud, Universidad Autónoma Metropolitana Iztapalapa, México City, México; ^3^ Instituto Nacional de Geriatría, S.S., México City, México; ^4^ Departamento de Patología Experimental, Instituto Nacional de Ciencias Médicas y Nutrición Salvador Zubirán, México City, México; ^5^ Institute of Pathology, University of Greifswald, Greifswald, Germany; ^6^ 1st Department of Medicine, University Medical Center, Johannes Gutenberg University Mainz, Mainz, Germany

**Keywords:** cholesterol, oxidative stress, ATM, DNA damage, carcinogenesis

## Abstract

Primary liver cancers represent the second leading cause of cancer-related deaths worldwide. Diverse etiological factors include chronic viral hepatitis, aflatoxin and alcohol exposure as well as aberrant liver lipid overload. Cholesterol has been identified as a key inducer of metabolic impairment, oxidative stress and promoter of cellular dysfunction. The aim of this work was to address the oxidative stress-mediated DNA damage induced by cholesterol overload, and its role in the development of hepatocellular carcinoma.

C57BL/6 male mice were fed with a high cholesterol diet, followed by a single dose of N-diethylnitrosamine (DEN, 10 μg/g, ip). Reactive oxygen species generation, DNA oxidation, antioxidant and DNA repair proteins were analyzed at different time points. Diet-induced cholesterol overload caused enhanced oxidative DNA damage in the liver and was associated with a decrease in key DNA repair genes as early as 7 days. Interestingly, we found a cell survival response, induced by cholesterol, judged by a decrement in Bax to Bcl2 ratio. Importantly, N-acetyl-cysteine supplementation significantly prevented DNA oxidation damage. Furthermore, at 8 months after DEN administration, tumor growth was significantly enhanced in mice under cholesterol diet in comparison to control animals. Together, these results suggest that cholesterol overload exerts an oxidative stress-mediated effects and promotes the development of liver cancer.

## INTRODUCTION

Aberrant lipogenesis has a major effect on many diseases, in addition to well-known metabolic disorders such as obesity and/or metabolic syndrome, lipid disorders also have crucial impact on tumor development. Lipids are essential building blocks, for new cellular membranes [1], but also are required for post-translational modifications in cell cycle-related proteins, such as Ras or heterotrimeric G proteins [2, 3], and are a major source of energy supply for cells [1]. Hepatocellular carcinoma (HCC), one of most frequent cancers globally, shows a remarkable dependence on sustained lipid synthesis [4]. Moreover, HCC aggressiveness is directly associated to the levels of lipogenic enzymes, related to fatty acid and cholesterol synthesis. Several reports have shown that increased activation of fatty acid synthase (FASN), ATP citrate lyase (ACLY), Acetyl-CoA carboxylase (ACC), mevalonate kinase (MVK), squalene synthase (SQS), and the 3-Hydroxy-3-methylglutaryl-CoA reductase (HMGCR), among others significantly drive the tumorigenic process and increases the aggressiveness of the tumors [5-7]. Although recent evidence supports the notion that exogenous free fatty acids play a major role in liver cancer development [8], the relevance of excessive dietary cholesterol in this context is less well understood. A recent report demonstrated that expression of HMGCR and MVK is significantly enriched in HCC human tissue when compared with the surrounding and normal liver tissue [6]. Furthermore, free cholesterol is increased in liver tissue from patients with non-alcoholic steatohepatitis (NASH), and this is associated to an increment in the expression of the sterol regulatory element binding protein (SREBP) 2 and steroidogenic acute regulatory protein (StAR) [9], both critically linked to HCC development and progression [10].

We and others have shown that cholesterol overload in the liver induces profound cellular redox imbalances, leading to the aggravation of different chronic liver diseases [11-14], predominantly by affecting mechanisms related to loss of glutathione (GSH) homeostasis in hepatocyte growth factor (HGF) and its receptor c-Met dependent manner [15, 16]. Oxidative stress is a well-addressed mechanism for DNA damage and carcinogenesis [17]. Therefore, redox imbalance can also enhance the carcinogenic effects of canonical DNA damage inducers such as *N*-nitrosodiethylamine (DEN) [18].

Given the chemical and biological properties of cholesterol and its effect for redox, we hypothesized that cholesterol can participate in the carcinogenic process, particularly by aggravating DNA damage in early stages of hepatocarcinogenesis. In the present work, we investigated the contribution of dietary cholesterol overload in the liver on chemically-induced DNA damage, and its effects on the redox status, antioxidant and DNA repair enzymes, to characterize a possible role of cholesterol as a tumor promoter in the liver.

## RESULTS

### Cholesterol overload enhances DEN-induced oxidative liver damage

**Figure 1:**
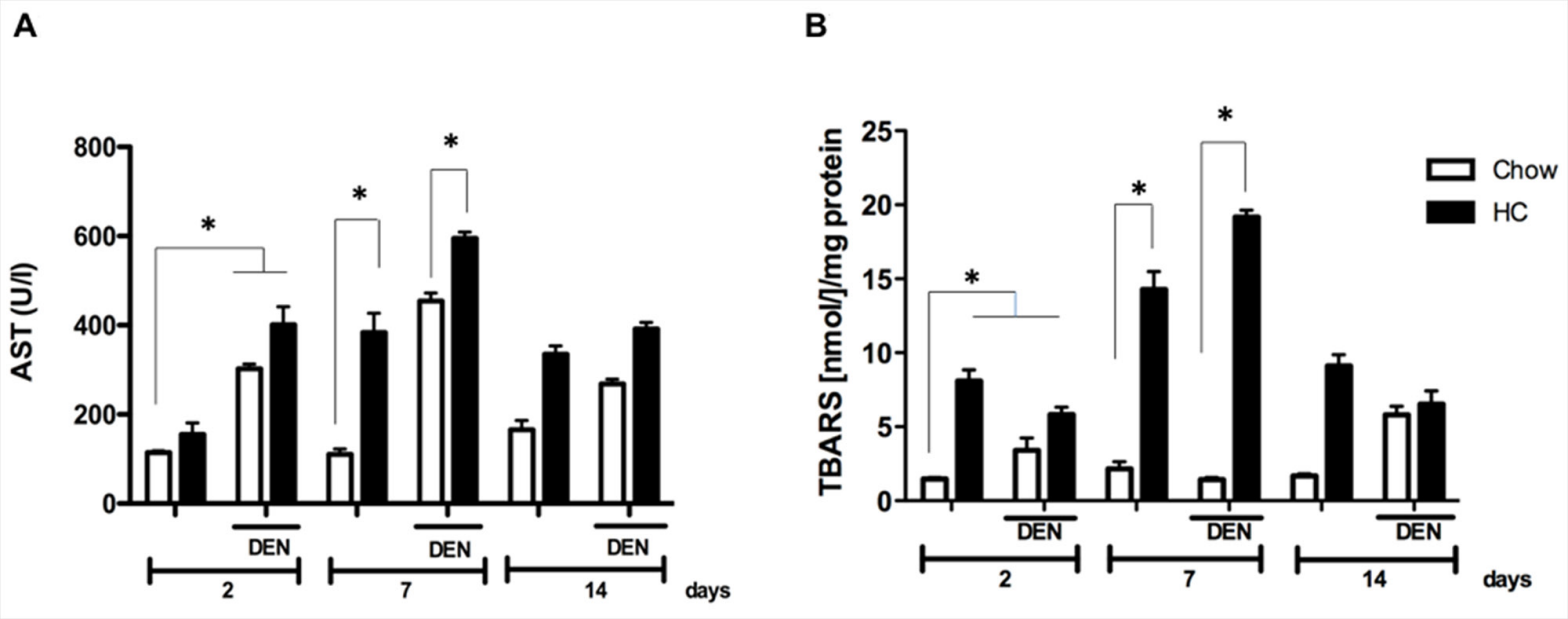
Cholesterol overload in the liver exacerbates the damage induced by *N*-nitrosodiethylamine Mice were subjected to both high cholesterol (HC) or Chow diet and two days later received, or not, a single dose of *N*-nitrosodiethylamine (DEN, 10 μg/g body weight), serum and liver tissue were recovered at 2, 7 and 14 days after DEN treatment. **(A)** Serum aspartate aminotransferase (AST) activity, and **(B)** lipid peroxidation determination assayed by thiobarbituric acid reactive substances (TBARS). Each column represents the mean ± SEM in at least five different mice. ^*^, *p* ≤ 0.05.

Given the well-known role of cholesterol as inducer of oxidative stress we, first addressed the extent of liver damage at 2, 7 and 14 days in all experimental groups. HC fed mice displayed a transient increase in AST serum levels starting two days under the treatment, peaking at 7 days when compared with animals under the CW diet and treated with DEN (DCW, Figure 1A). Interestingly, AST activity decreased after 14 days of treatment, suggesting liver healing response after the acute damage.

**Figure 2:**
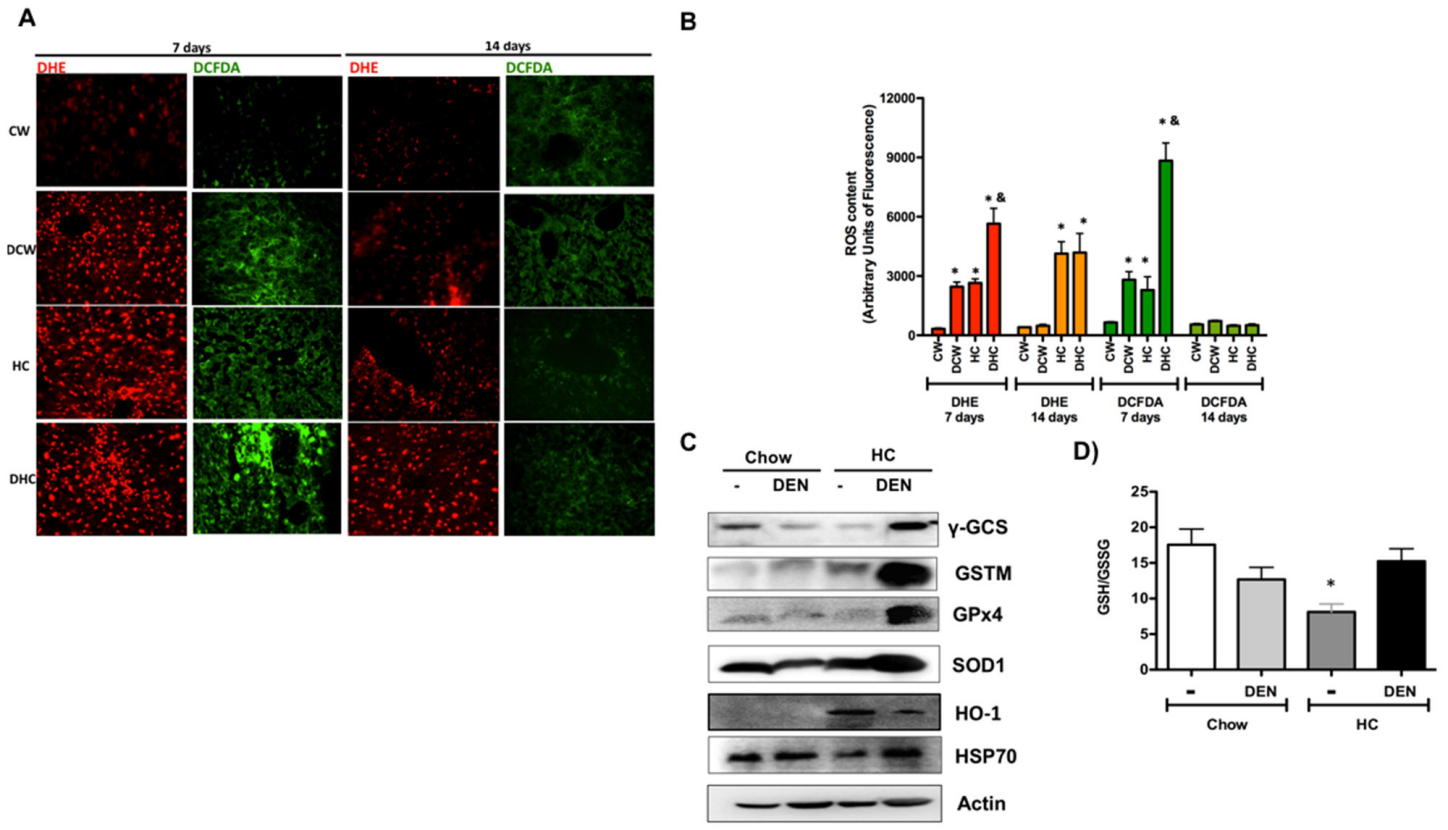
Cholesterol overload in the liver disrupt redox homeostasis **(A)** Reactive oxygen species content in liver tissue were determined by dihydroethidium (DHE, 5 μM, in red) and 2’,7’ –dichlorofluorescin diacetate (DCFDA, 5 μM, in green). Representative images obtained by confocal microscopy. Original magnification X100. **(B)** Fluorescence quantification of ROS determination. **(C)** Western blot of the main antioxidant and protective proteins at day 7. Images are representative of at least three independent experiments. Actin was used as loading control. **(D)** Determination of reduced glutathione (GSH) and oxidized GSH (GSSG) in livers at day 7. Each column represents the mean ± SEM in at least four different mice. ^*^, *p* ≤ 0.05 vs respective Chow samples at the same time. &, *p* ≤ 0.05 vs Chow-DEN at the same time.

To address the degree of oxidative damage, lipid peroxidation was determined in whole liver tissue. In accordance to the increased activity of AST, levels of TBARS were significantly increased at 7 days in animals under HC diet, (7-fold *vs* CW and DCW), returning to similar levels observed at day 2 at day 14 (Figure 2B). Data clearly show a maximal damage response at 7 days.

To figure out the ROS involvement in cholesterol-mediated liver damage, we addressed both, superoxide radical (O_2_^-·^), by DHE; and peroxides, such as H_2_O_2_, by DCFDA fluorescence, in fresh liver sections at 7 and 14 days. Representative confocal images of ROS determination are shown in Figure 2. It is of notice that the HC diet alone increases both O_2_^-·^ and peroxides cellular content at 7 days, approximately 8-fold versus CW, which is related to TBARS content and AST activity. ROS content was exacerbated in DEN-treated HC group, indicating a greater loss of redox homeostasis. ROS content was significantly diminished at 14 days, confirming that day 7 is the time for maximal oxidative stress generation (Figure 2B).

The assessment of some of the main protective and antioxidant proteins revealed an adaptive response in DHC mice (Figure 2C). Remarkably, the levels of GSH-related enzymes such as γ-GCS, GSTM and GPx4 increased in the DHC group (densitometric analysis in Supplementary Figure 2), suggesting that GSH system is required for this kind of damage as we and others previously reported [11, 14, 18]. Interestingly, the GSH to GSSG ratio determined at day 7 exhibited a significant decrease in livers from HC fed animals, but HC and DEN treated mice show similar values of GSH than control Chow (Figure 2D), this finding agrees with γ-GCS content observed in HC-DEN group, other antioxidant and protective enzymes such as SOD1, HO-1 and HSP70 were also increased in this group comparing with Chow and Chow-DEN animals.

**Figure 3:**
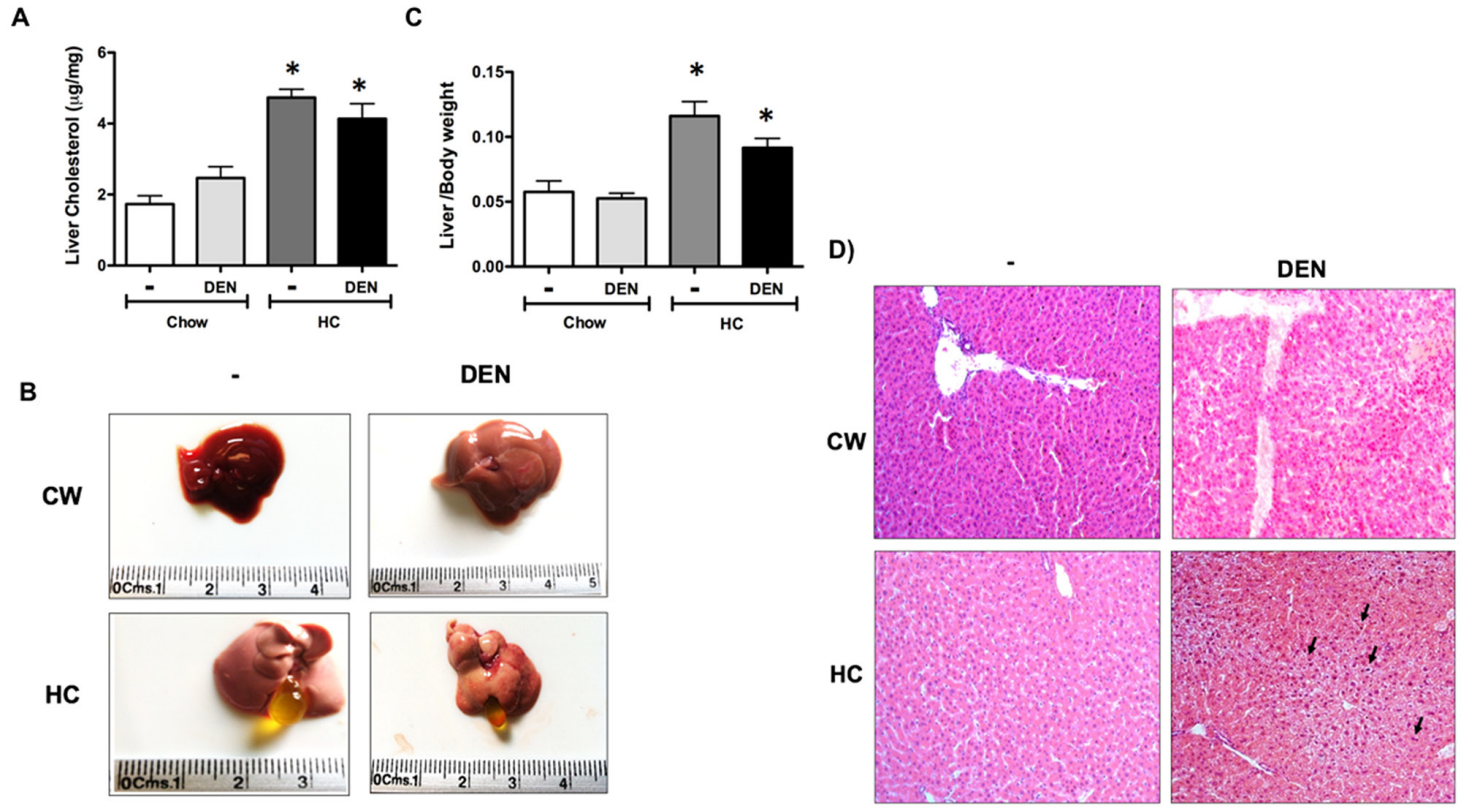
Macroscopic and microscopic inspection of the liver under HC diet and DEN treatment **(A)** Cholesterol content in liver tissue. **(B)** Macroscopic aspect of the liver from different groups. **(C)** Liver to body weight ratio. Each column represents the mean ± SEM in at least four different mice. ^*^, *p* ≤ 0.05 vs Chow control animals. **(D)** Histology appearance by H&E staining, arrows show cells with mitogenic activity corroborated by chromatin distended nuclei. Images are representative of at least 4 different mice, original magnification X200.

Since effects were most striking at 7 days, we measured total cholesterol levels in liver tissue from animals of all groups. Figure 3A shows that mice fed with HC diet certainly exhibited significant cholesterol increment. Macroscopic inspection of livers shows the characteristic steatotic pale color in HC fed animals (Figure 3B). Gallbladder hypertrophy was also observed in HC-fed animals, and these characteristics were related to a slight, but significant, increment in liver to body weight ratio in both groups under HC diet (Figure 3C).

Histology analysis revealed multivesicular steatosis in both groups under HC diet, with the DEN treated group exhibiting a mild inflammatory infiltration. Mitogenic activity was also corroborated by the observation of chromatin distended nuclei (arrow), nucleoli acidophilic staining, a key indication of cellular metabolism activation and constant rRNA synthesis (Figure 3D).

### HC promotes an antiapoptotic response and DNA damage

**Figure 4:**
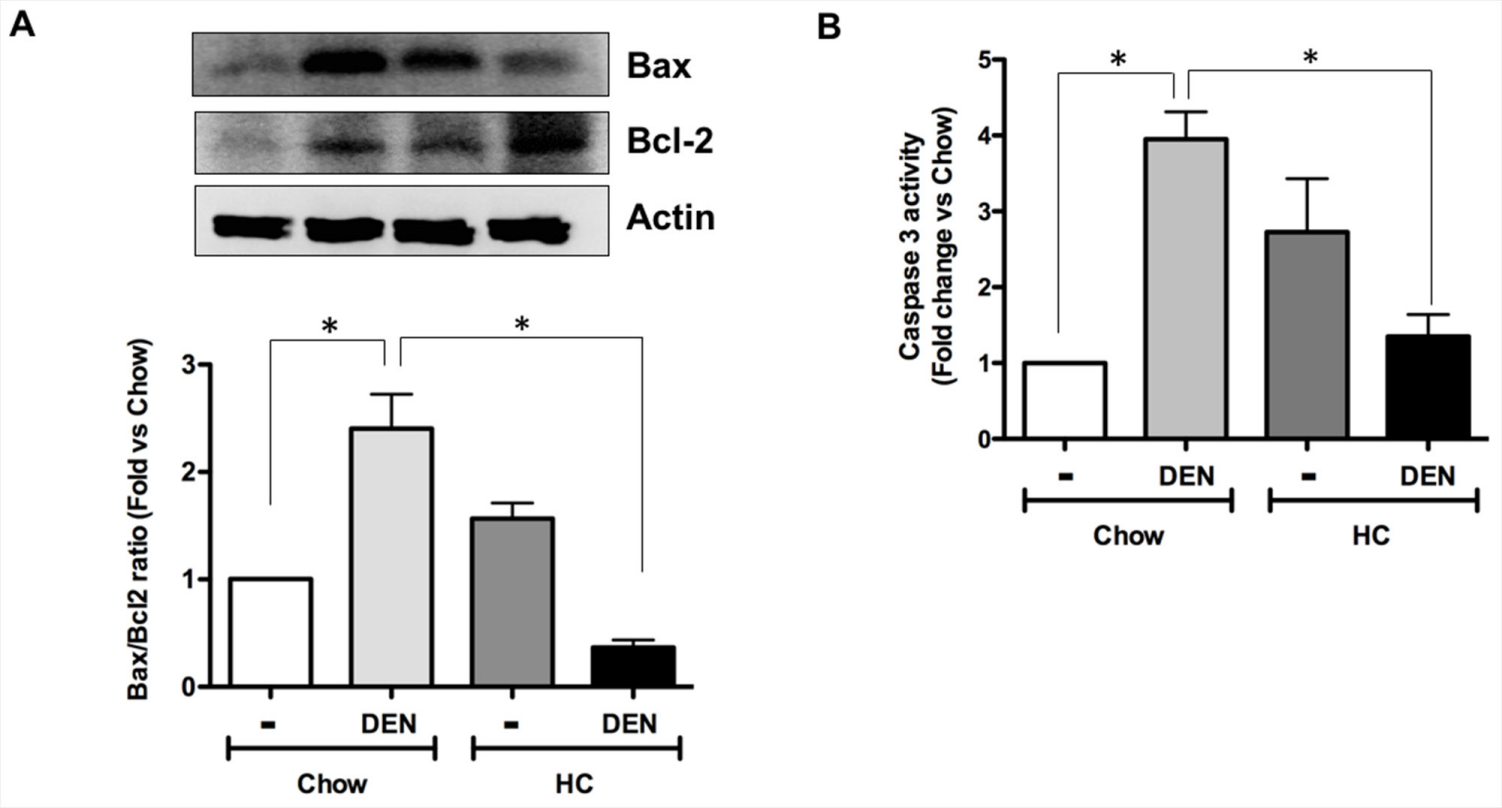
HC promotes an antiapoptotic response and DNA damage **(A)** Bax to Bcl2 ratio of protein content determined by Western blot in samples from all animal groups, representative images of at least three different mice. Actin was used as loading control. **(B)** Caspase 3 activity in whole tissue homogenates. Each column represents the mean ± SEM in at least four different mice. ^*^, *p* ≤ 0.05.

Although there is an increment in antioxidants enzymes and GSH in animals under HC diet and DEN treatment, ROS content is significantly increased as well, suggesting an inefficient antioxidant response. This sustained pro-oxidant environment could promote apoptosis. To assess the apoptosis status in the respective groups, we assayed Bax to Bcl2 expression ratio in the livers. As expected, DEN treatment led to a significant increase in the ratio in animals treated with chow diet. However, HC fed animals treated with DEN showed a striking activation of Bcl-2 and concomitant downregulation of Bax (Figure 4A). Apoptosis was corroborated by caspase 3 activity in whole liver homogenate (Figure 4B) observing a decrease in caspase 3 activity in HC fed animals treated with DEN comparing with Chow-DEN. Thus, these data suggest that the HC diet induces apoptosis resistance that might facilitate a cell transformation due to an unresolved cell damage.

### Cholesterol overload in the liver enhances the DNA damage induced by DEN and impairs DNA repair-related proteins activation

**Figure 5:**
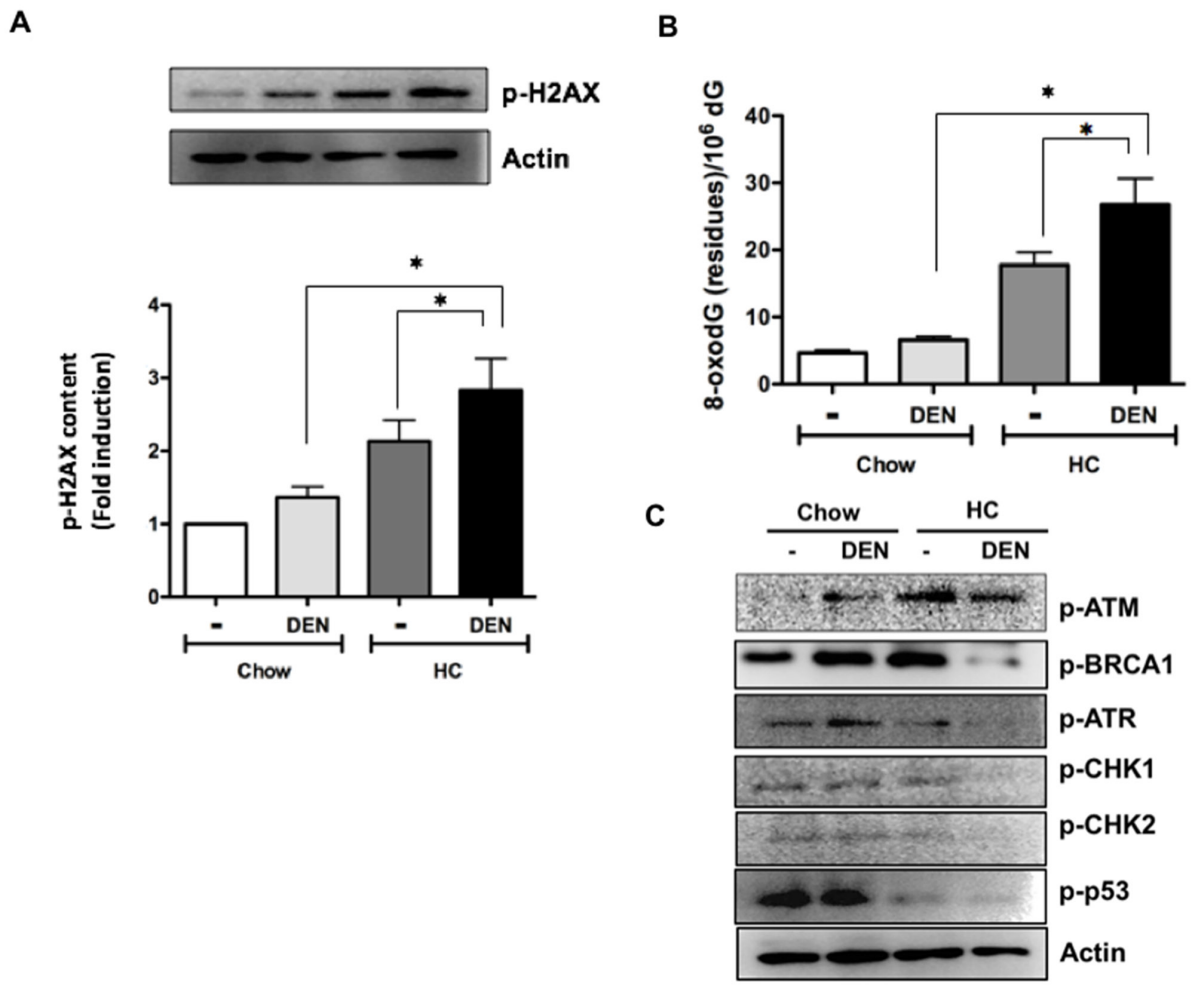
Cholesterol overload in the liver potentiates the DNA damage induced by DEN impairing DNA repair enzymes activation **(A)** Phosphorylation of histone 2AX (p-H2AX) determined by Western blot. **(B)** Guanine oxidation judged by 8-oxodeoxiGuanine (8-oxodG) determined by HPLC. **(C)** Representative images of Western blot analysis of the main proteins related to DNA repair. Actin was used as loading control. Each column represents the mean ± SEM in at least four different mice. ^*^, *p* ≤ 0.05.

Next, we determined the activation of the histone 2AX (H2AX), a surrogate marker of DNA damage, in samples from 7 days of treatment. Consistent with the increased liver damage and ROS activation, Figure 5A shows an increment in the phosphorylation of the H2AX in HC groups, particularly in those treated with DEN, damage was corroborated by measuring the deoxyguanosine (dG) oxidation into 8-oxo-dG, another key marker of DNA oxidation. Of note, at 7 days HC fed animals showed a significant increase in 8-oxodG (Figure 5B), particularly in those treated with DEN.

To dissect the underlying molecular mechanism, we evaluated the levels of key proteins involved in DNA repair. Figure 5C shows representative immunoblot images of p-ATM, p-BRCA1, p-ATR, p-CHK1, p-CHK2 and p-p53; importantly, all proteins were significantly decreased in DHC mice, whereas p-ATM increased in Chow fed animals (densitometric analysis in Supplementary Figure 3).

### NAC treatment counteracts ROS generation and the oxidative DNA damage

**Figure 6:**
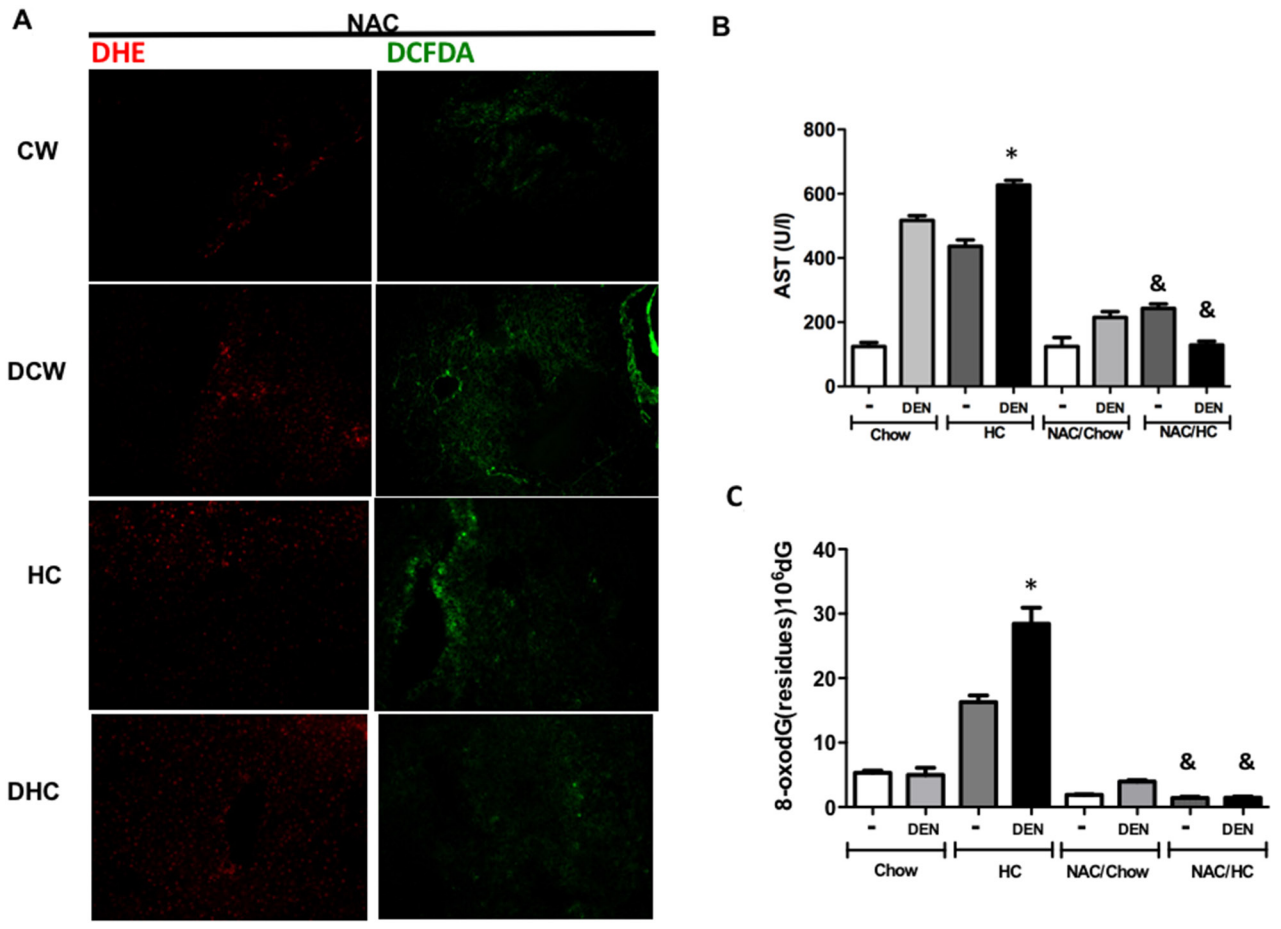
N-acetylcysteine prevents oxidative stress and DNA oxidative damage A cohort of animals treated for 7 days received 80 mmol/L N–acetyl–L-cysteine (NAC) in drinking water *ad libitum*. **(A)** Reactive oxygen species content in liver tissue were determined by dihydroethidium (DHE, 5 μM, in red) and 2’,7’ –dichlorofluorescin diacetate (DCFDA, 5 μM, in green). Representative images obtained by confocal microscopy. Original magnification X100. **(B)** Serum aspartate aminotransferase (AST) activity, **(C)** 8-oxodeoxiGuanine (8-oxodG) content determined by HPLC. Each column represents the mean ± SEM in at least four different mice. ^*^, *p* ≤ 0.05 vs Chow control animals; &, *p* ≤ 0.05 vs DEN treated Chow.

The disruption of cellular redox status aggravates the harmful effects mediated by DEN, and our data clearly show an impairment of the GSH system. To elucidate whether the redox homeostasis could be re-established we added NAC (80 mmol/L) in drinking water along HC diet for 7 days. As expected, NAC significantly decreases ROS content in all conditions, particularly in those under HC diet and DEN treatment (Figure 6A and Supplementary Figure 1), while concomitantly leading to an improvement in liver function (Figure 6B) and a decrease in DNA damage judged by dG oxidation (Figure 6C).

### A HC diet promotes tumor progression

**Figure 7:**
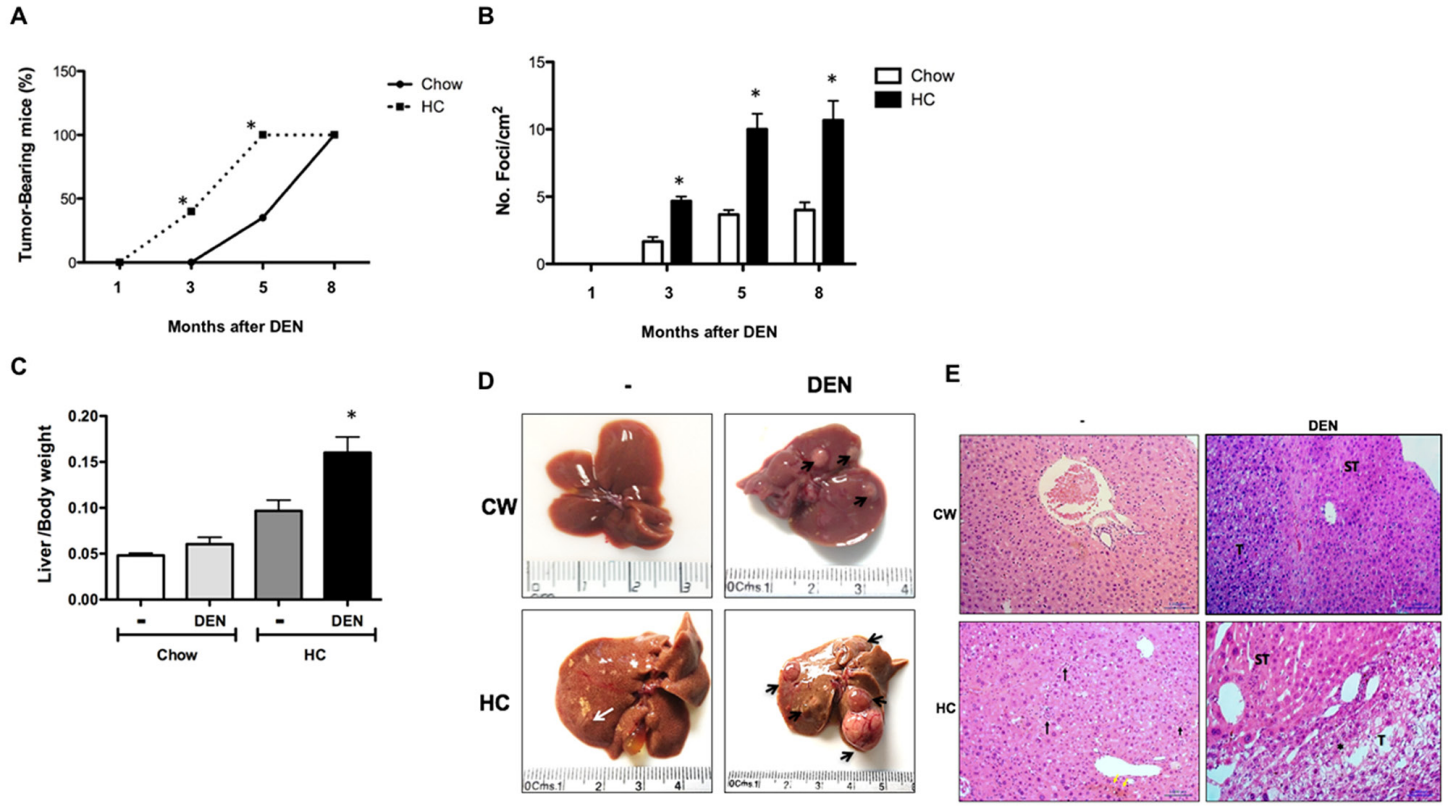
Mice subjected to HC diet are more susceptible than Chow mice to DEN-induced hepatocarcinogenesis **(A)** Incidence and time course of hepatic lesions from 1 to 8 months after DEN treatment, **(B)** multiplicity of foci per square centimeter. **(C)** Liver to body weight ratio at month 8. Each column or point represent the mean ± SEM in at least four different mice. ^*^, *p* ≤ 0.05 vs Chow control animals; &, *p* ≤ 0.05 vs DEN treated Chow, **(D)** Macroscopic inspection of the livers at month 8, **(E)** Histology appearance by H&E staining, perilobular, periportal inflammation (black arrow), cholestasis (yellow arrow), inflammation (·), tumor (T), surrounding tissue (ST). Images are representative of at least 4 different mice, original magnification X200.

To address if the exacerbated DNA damage observed in DHC could be associated to progression of HCC and an aggressive phenotype, we explored the different investigated groups at 1, 3, 5 and 8 months as we previously reported [18]. The kinetics of tumor development was assessed by incidence and multiplicity. In agreement with our findings from the acute model, chronic cholesterol overload in the liver led to a more rapid development of tumors than in DCW mice (Figure 7A). Indeed, at 3 months 44 % (4 of 9, p ≤ 0.05) of DHC mice developed tumors comparing with 0% in DCW mice, at 5 months all DHC animals developed tumors versus 40% of DCW. The tumor incidence in DHC mice was higher compared with DCW at 3, 5 and 8 months after DEN treatment (Figure 7B). At 8 months of treatment, the liver to body weight ratio (Figure 7C) was higher in DHC animals comparing with HC and CW animals, and the data agree with the incidence and multiplicity of the lesions. Gross liver inspection of DHC livers revealed multiple tumors (Black arrow), bigger and evidently, more vascularized than those seen in DCW. Small lesions were observed in HC livers (white arrow).

Additionally, histological analysis of HC tissue (Figure 7E), revealed steatohepatitis, picnotic nuclei and increased mitotic activity, perilobular, periportal inflammation (black arrow), and signs of cholestasis (yellow arrow). All the observed cells by the field were identified as neoplastic due the loss of nucleus/cytoplasm ratio, granular cytoplasm that indicates massive mitochondrial presence and more than five nucleoli per nucleus. Along with these cellular alterations, the liver normal architecture was completely lost. DHC livers exhibited multi-vesicular steatosis, increased mitotic activity, that extensively describes a well-differentiated carcinoma. Hepatocytes displayed acidophilic nuclei, loss of liver normal architecture and perilobular and periportal inflammation (·), it is clear the difference between the transformed cells that conform the tumor (T) when compared with those on the surrounding tissue (ST). The tumor cellular changes observed in this tissue were much more dramatic than those found in DCW treated liver sections. Interestingly, we found metastasis in lungs only in some animals (5 of 9) under HC diet and DEN treatment (Supplementary Figure 4).

## DISCUSSION

Aberrant lipogenesis is a critical event in many pathologies, such as obesity, metabolic syndrome, some viral infections, among others [19]. Increasing evidence supports that, in cancer, lipogenesis is overacting to support the tumor growth [20], because lipids are required as fuel, building blocks for new cellular membranes, and some lipid intermediaries are essential for post-translational modifications in key proteins such as Ras or heterotrimeric G proteins [2, 3]. Particularly, in human HCC, Calvisi and coworkers, outstandingly proved that aberrant lipogenesis is directly associated to an aggressive phenotype and poor prognosis [6]. The expression of key proteins associated with lipogenesis was found to be increased in HCC, being higher as HCC malignancy progress. Among the proteins correlated with a poorer outcome are the fatty acid synthase (FASN), acetyl-coenzyme A carboxylase, and the overactivation of their main transcription factor, such as SREBP 1. Remarkably, those related to mevalonate pathway were also increased such as mevalonate kinase, HMGCR, and the master transcription factor SREBP 2, suggesting that cholesterol could be positioned as a key element in HCC progression. We were focused to figure out if cholesterol plays determinant role in DNA damage and HCC initiation.

We and others have been proved that the consumption of a high cholesterol diet induces a cholesterol overload in the liver rising a complex response in hepatocytes characterized by mitochondria dysfunction and oxidative stress [12-14], but, interestingly, hepatocytes with cholesterol overload seems to be resistant to cell death under unstimulated conditions. Nonetheless, they are sensitized to a second aggression, particularly those directed to mitochondria by a mechanism related to mitochondrial-glutathione depletion [14]. In DHC mice, we found that GSH-related enzymes were remarkably increased (Figure 2C) perhaps as a compensatory response, because GSH levels are considerably decreased in hepatocytes with cholesterol overload as we and others have demonstrated [11, 13, 14], interestingly DHC tissue exhibited GSH levels compared as Chow control animals (Figure 2D), suggesting that both harmful stimulus, cholesterol and DEN display different regulatory pathways directed to control redox homeostasis. GPx4 is a mitochondria-resident antioxidant enzyme directed to reduce hydrogen peroxides and lipid hydroperoxides, and the enzyme is fundamental for redox mitochondria homeostasis [21]. It is relevant that GPx4 is overexpressed in DHC tissue, recently, Guerriero and coworkers [22] showed that the overexpression of GPx4 is correlated to malignancy, exhibiting a higher expression in HCC grade III comparing with I and II.

Oxidative stress is the leading mechanism of damage in the liver with high cholesterol content as we have confirmed in the present work (Figure 1 and 2). However, exploration of some markers of cell death revealed that cells seem to be resistant to death as judged by the Bax to Bcl2 ratio and caspase 3 activity (Figure 4). It has been reported that mitochondria isolated from HCC exhibit high levels of cholesterol, resistance to mitochondrial membrane permeabilization, and release of proapoptotic mediators such as cytochrome c and Smac/Diablo, being resistance to Bax activity [23], these characteristics confers an aggressive phenotype and contributes to chemotherapy resistance. Although, cholesterol overload has been associated to apoptosis resistance, we gain evidence that, part, such response could be mediated by Bcl2 expression when liver is in presence of DEN (Figure 4A), this was related to a decrease in Bax content indicating a clear antiapoptotic effect corroborated by caspase 3 activity. Under HC diet, mitochondrial cholesterol overload [14] disturbing metabolism and impairing mitochondrial membrane dynamic [24], this could explain the antiapoptotic effect, profounder attention requires the cholesterol-mediated Bcl2 expression to exploit the potential therapeutic intervention.

ROS are the main inducers of DNA damage and, consequently, of mutations. Indeed, particularly mitochondria-derived ROS have been implicated to initiation, progression and aggressiveness of cancer [25-27]. Figure 5 shows the increment of two well-documented markers of DNA damage. The level of 8-oxodG in plasma, urine, and cerebrospinal fluids has been used as marker of DNA damage [28]. In addition, the formation of 8-oxodG is linked to epigenetic instability in human HCC [29], and it has been identified as a risk factor for the development of HCC in patients with chronic hepatitis C virus infections. In addition, it has been suggested that these patients must be monitored carefully [30]. Taking into consideration the confidence on 8-oxodG determination as a good marker of DNA damage, our findings are remarkable indicating a broad damage induced by DEN in an hypercholesterolemic environment. Even more, activation of the H2AX, another excellent marker for DNA damage in humans [31], confirms the exacerbation of the genomic damage promoted by the high cholesterol liver content and DEN treatment.

The extensive DNA damage triggers a mechanism named DNA damage response (DDR), which detects and repairs DNA by inducing cell cycle arrest to guarantee that only cells in good conditions can progress and proliferate. Some reports indicate that activation of DDR proteins can be increased during early stages of tumorigenesis [32, 33]. It has been suggested that this response can work as a barrier for tumor development, but this is dependent of p53 activation [34]. In our system, as Figure 5D shows, p53 phosphorylation is practically abrogated in animals under HC diet, suggesting the progression of aberrant cells into the cell cycle conditioning to tumorigenesis. The results also indicate that, although ataxia-telangiectasia mutated (ATM) activation is increased in HC tissue, in DHC is downregulated but not abrogated as the other repair enzymes are. It seems that the ATM-initiated signal transduction is partially impaired due to one of the main downstream effectors, CHK2, which is decreased only in DHC tissue.

ATM is primarily activated by double-stranded DNA breaks (DSB), but ATR also responds to a broad spectrum of damages [35]. We have the evidence that cholesterol overload in the liver induces DNA oxidation as judged by 8-oxodG, and this triggers ATM activation. This supports previous findings that ATM and oxidative stress are related each other and, particularly, ATM seems to display protective effects [36]. Accordingly, our data clearly indicate a relation of ATM levels with a protective effect (Figure 5D). HA2X is a downstream substrate of ATM leading to the enhancement of the DDR, confirming that in high cholesterol-induced DNA damage ATM is leading the protective response.

We have previously reported that NAC supplementation can prevent carcinogenic effects of oxidative stress [18], and in the present work we confirmed once again this relevant property of NAC (Figure 6). It has been reported that ATM^-/-^ cells are under oxidative stress and an increment in 8-oxodG has been observed, this condition was reverted by NAC treatment counteracting genetic instability [36, 37].

Our work offers the mechanism driving the enhancement of a canonical carcinogenic effect by cholesterol overload in the liver, which can promote a faster development of tumors (Figure 7) with an aggressive phenotype, judged by lung metastasis (Supplementary Figure 4), in fact, lipid metabolism has extensively been related to cancer metastasis [38], and it has recently been reported that cholesterol metabolism plays key role in metastasis in an experimental model of pancreatic cancer, the abrogation acyl-CoA cholesterol acyl transferase-1 (ACAT-1), significantly suppresses tumor growth and metastasis [39], even more, the use of simvastatin, an inhibitor of cholesterol synthesis, blocks the transforming growth factor beta-induced epithelial-mesenchymal transition, a hallmark for cancer metastasis [40], our data, leave clear the relevance of cholesterol content in cancer metastasis.

The assessment of the activation of ATM and HA2X, and the content of GPx4 and GSTM in early liver lesions could represent good prognosis factors for aggressive presentations of HCC, although many reports indicate that cancer therapies with antioxidants do not provide benefits, but detriments in some cases, definitively in carcinogenesis processes antioxidants could be fundamental, particularly those serving as precursors for endogenous antioxidants synthesis such as NAC, that cells can manages properly as we previously demonstrated [18] and confirmed in the present work.

Data confirm that cholesterol can play a relevant role not only in HCC progression but in cell transformation, by a mechanism related to the increment of ROS levels and apoptosis decrement at early times that lead to DNA damage and initiation of carcinogenesis. Our findings are in agreement with data published by Dr. Calvisi and colleagues [6], indicating that cholesterol homeostasis-related proteins are overexpressed in HCC with poor prognosis, even more RNA-seq analysis of human HCC revealed disorders in lipid metabolism, metabolism of xenobiotics, and ATM signaling [41], some genomic reports also confirm that our mouse data are relevant in human, particularly those related with oxidative stress, DNA damage and lipid metabolism [42]. Our findings are positioning cholesterol cellular overload in the liver as a tumor promoter, inducing oxidative stress, impairing DNA damage repair system and promoting and accelerated tumor progression (Supplementary Figure 5). Hypercholesterolemia should be closely monitored in those patients with HCC risk factors.

## MATERIALS AND METHODS

### Animals

Sixty 14-days old male mice (C57/BL6) were randomly separated in four groups: i.) fed with a high cholesterol diet (HC, 2% cholesterol and 0.5% sodium cholate) as previously reported [11, 13, 14]; ii.) HC diet and a single intraperitoneal (ip) injection of 10 μg/g body weight of DEN (Sigma-Aldrich) as previously reported [18]; control animals received regular Chow diet (CW) with iii.) or without iv.) DEN. An extra cohort of mice fed HC or Chow diet for 7 days and treated or not with DEN, received 80 mmol/L N–acetyl–L-cysteine (NAC, Sigma-Aldrich) in drinking water *ad libitum*.

Mice were euthanized and serum and liver tissue were examined at 2, 7 or 14 days and 1, 3, 5 and 8 months. All animals were maintained in specific pathogen-free housing and cared in accordance with the NIH Guide for the Care and Use of Laboratory Animals.

### Histology

Livers were fixed in 10% neutral formalin overnight at 4°C. Paraffin sections (5 μm) were stained with routine H&E for histology and quantification of liver lesions (foci). Analysis was conducted by an expert pathologist (R.H.P.).

### Western blot

Western blot was performed as we previously reported [43], using specific antibodies listed in Supplementary Table 1.

### Lipid peroxidation

Lipid peroxidation was assayed by the production of thiobarbituric acid reactive substances (TBARS) using spectrophotometry as described by Buege and Aust [44].

### DNA extraction, enzymatic hydrolysis, and analysis of 8-oxo-7,8-dihydro-2’-deoxyguanosine (8-oxodG) by HPLC/electrochemical detection

DNA was extracted from fresh liver samples using the chaotropic-NaI method, as we previously reported [45]. After DNA quantification using Nanodrop 2000c (Thermo Scientific Inc), samples were digested with nuclease P1 and *E. coli* acid phosphatase. 8-oxodG detection was performed as we reported previously [45]. Briefly, 100 μg of digested DNA was loaded into a HPLC coupled with an electrochemical detector (EC, Waters Inc) and the system was connected to a Supelcosil LC-18 (Supelco, Bellefonte, PA, USA) reverse-phase column (250X4.6 mm, i.d. particle size 5 μm). The eluent was 50mM potassium phosphate buffer, pH 5.5, with 8% methanol at 1 ml/min flow rate. The molar ratio of 8-oxodG in each DNA sample was determined based on EC detection at 290 mV for 8-oxodG and absorbance at 254 nm for dG.

### ROS in situ determination

Animals were euthanized in parallel exclusively for *in situ* ROS determination [12]. Fresh tissue was rapidly sectioned, frozen in liquid nitrogen, and embedded in optimum cutting temperature reagent (OCT, Sakura Finetec, Torrance, CA). Subsequently, 8-μm frozen sections were obtained in a cryostat (Leica CM-3050S) at −20°C and the slides were immediately incubated for 15 min, in the dark, at room temperature with either 2’,7’ –dichlorofluorescin diacetate (DCFDA, 5 μM), a cell-permeable non-fluorescent probe that is intracellularly de-esterified and converted to the highly fluorescent 2′,7′-dichlorofluorescein upon oxidation by ROS, particularly peroxides, or with dihydroethidium (DHE, 5 μM) for determination of superoxide anion radical detecting ethidium fluorescence. Samples were covered and observed using a confocal microscope at excitation and emission wavelengths of 480 and 520 nm, respectively, for DCFDA; and excitation and emission wavelengths of 485 and 570 nm, respectively, for DHE-derived ethidium fluorescence, as we previously reported [46].

### Caspase 3 activity

Caspase 3 activity was quantified using the caspase 3 synthetic fluorogenic tetrapeptide substrate Ac-DEVD-AMC (BD Pharmingen) as previously we reported [12] using fresh liver tissue, values were normalized regarding Chow sample and reported as fold change.

### Serum detection of alanine aminotransferase activity

Blood samples were obtained from each animal. Serum levels of alanine aminotransferase (ALT) were determined by the automated method using Reflovet Plus (Roche).

### Glutathione determination

Both reduced and oxidized glutathione (GSH and GSSG, respectively) were determined using the Glutathione assay kit (Sigma-Aldrich # CS0260) following manufacturer´s instructions. Values are reported as GSH to GSSG ratio.

### Protein content

Protein content was evaluated by the bicinchoninic acid protein assay (BCA) kit following the manufacturer´s instructions (Thermo Scientific).

### Statistical analysis

Data are presented as mean ± SEM of at least three independent experiments carried out in triplicate. Comparisons between groups were made using Student’s *t* test, Mann Whitney and Tukey-Kramer test. GraphPad Prism 6 software for OSX was used to run analysis. Differences were considered significant at *p*≤ 0.05.

## SUPPLEMENTARY MATERIALS FIGURES AND TABLE



## Abbreviations

DEN: N-diethylnitrosamine
HC: high cholesterol
CW: Chow regular diet
ROS: reactive oxygen species
HCC: hepatocellular Carcinoma
FASN: fatty acid synthase
ACLY: ATP citrate lyase
ACC: Acetyl-CoA carboxylase
MVK: mevalonate kinase
SQS: squalene synthase
HMGCR: the 3-hydroxy-3-methylglutaryl-CoA reductase
SREBP: sterol regulatory element binding protein
GSH: reduced glutathione
GSSG: oxidized glutathione
AST: aspartate aminotransferase
TBARS: thiobarbituric acid reactive substances
8-oxodG: 8-oxo-7,8-dihydro-2´-deoxyguanosine
DCFDA: 2´,7´-dichlorofluorescein diacetate
DHE: dihydroethidium
NAC: N-acetyl-L-cysteine
