# Supplementary material for: Cholesterol overload in the liver aggravates oxidative stress-mediated DNA damage and accelerates hepatocarcinogenesis
Source: Oncotarget. 2017 Oct 24;8(61):104136–48. doi: 10.18632/oncotarget.22024 (PMC5732793; doi:10.18632/oncotarget.22024)
Supplement: Supplementary file 1 [file oncotarget-08-104136-s001.pdf]

Cholesterol overload in the liver aggravates oxidative stress-mediated DNA damage and accelerates hepatocarcinogenesis

SUPPLEMENTARY MATERIALS

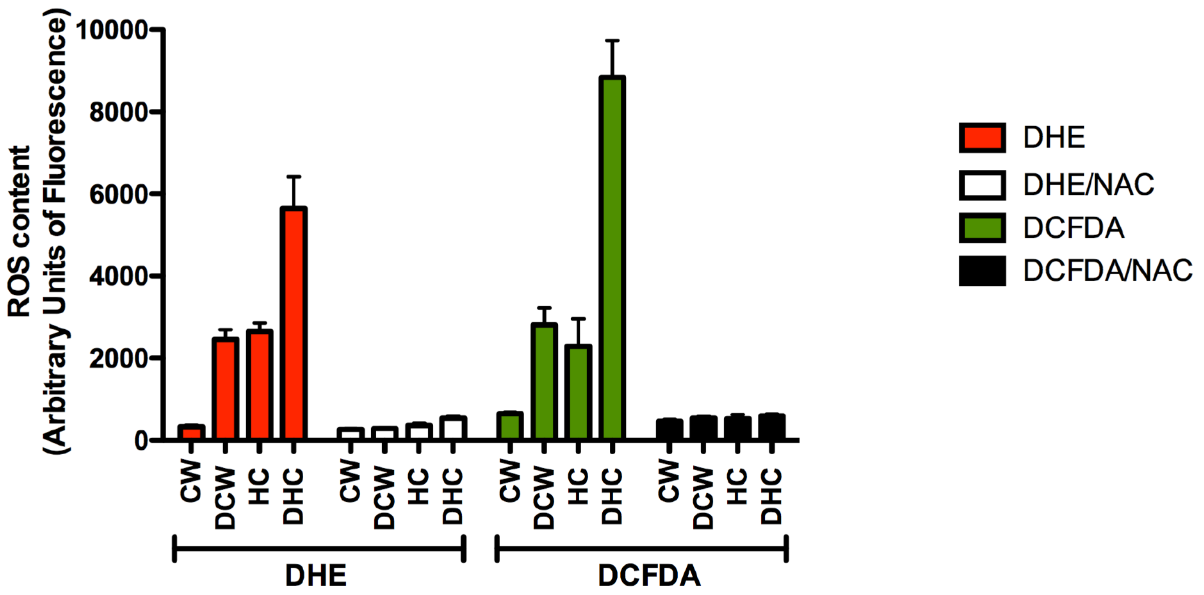

Supplementary Figure 1: Reactive oxygen species quantification in liver tissue. ROS determine at day 7 by dihydroethidium (DHE, 5 µM) and 2',7' –dichlorofluorescin diacetate (DCFDA, 5 µM) in presence or abscent of NAC. Each column represents the mean ± SEM in at least four different mice.

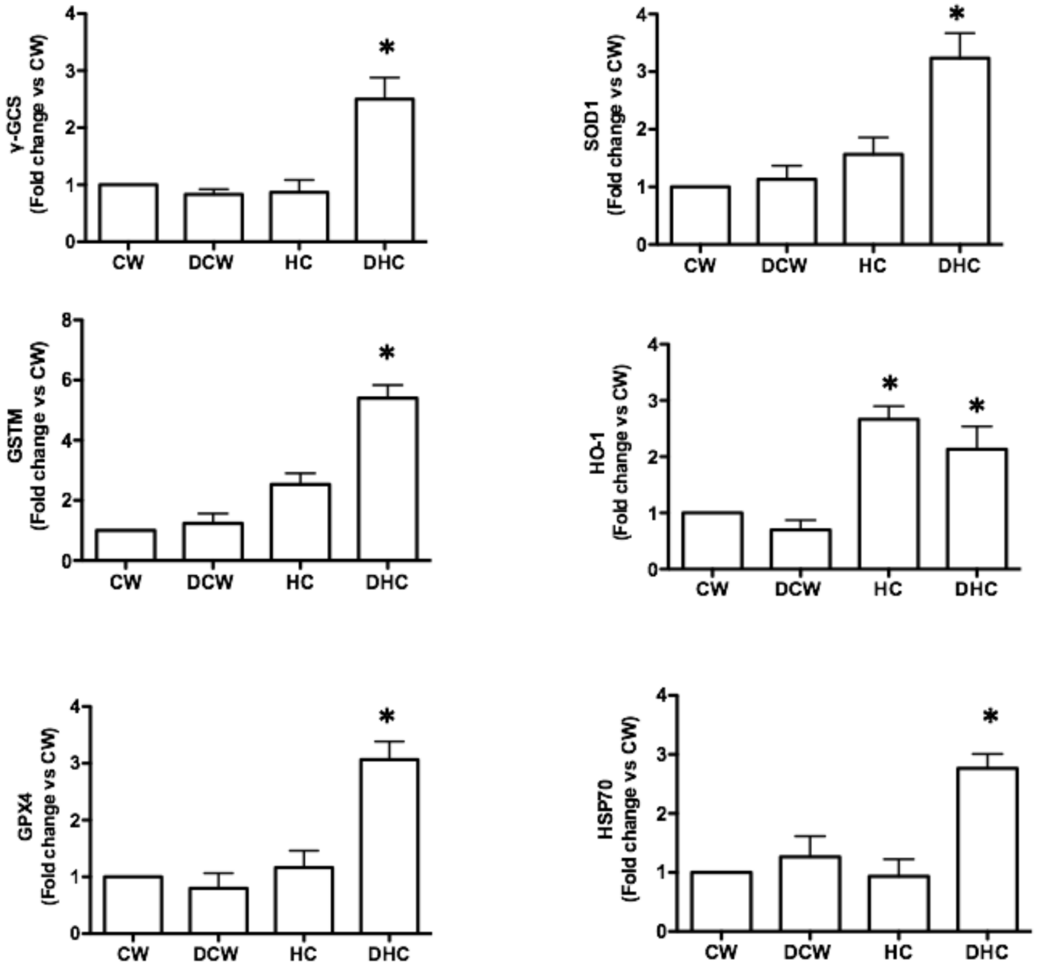

Supplementary Figure 2: Densitometric analysis of antioxidant and protective proteins. Each column represents the mean ± SEM in at least four different mice. *, $p \leq 0.05$ vs Chow fed animals (CW).

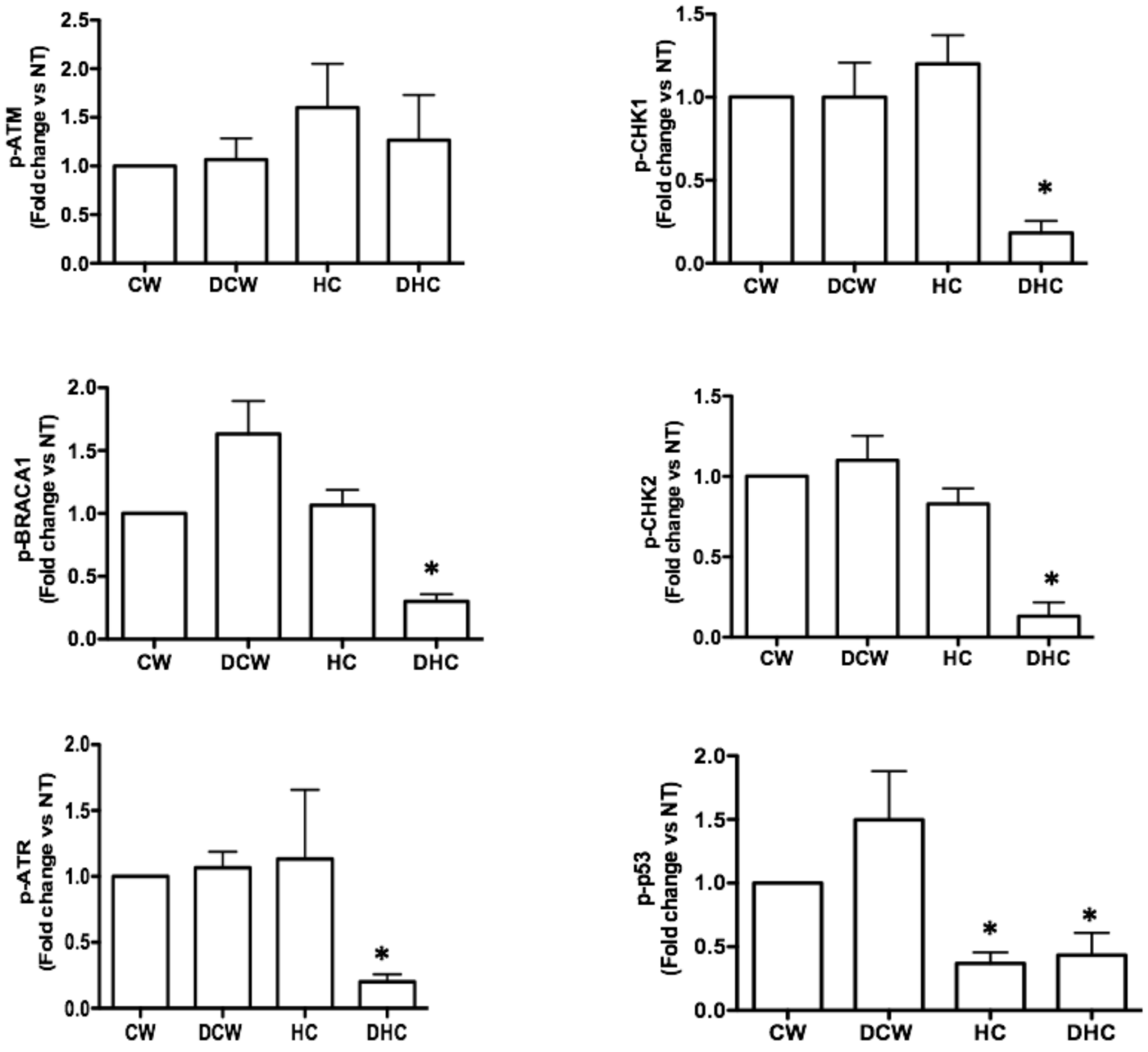

Supplementary Figure 3: Densitometric analysis of DNA repair-related enzymes. Each column represents the mean ± SEM in at least four different mice. *, $p \leq 0.05$ vs Chow fed animals (CW).

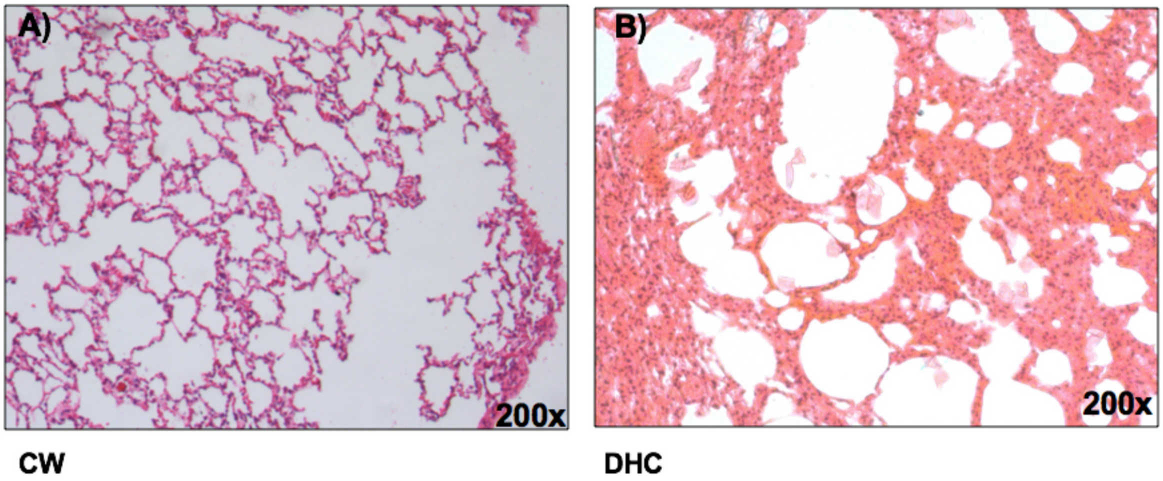

Supplementary Figure 4: DEN treatment induces lung metastasis under HC diet at month 8. Histology analysis by H&E routine staining of lungs from **(A)** Control Chow (CW) animals; **(B)** DEN treated mice under high cholesterol (HC) diet exhibiting the metastasis.

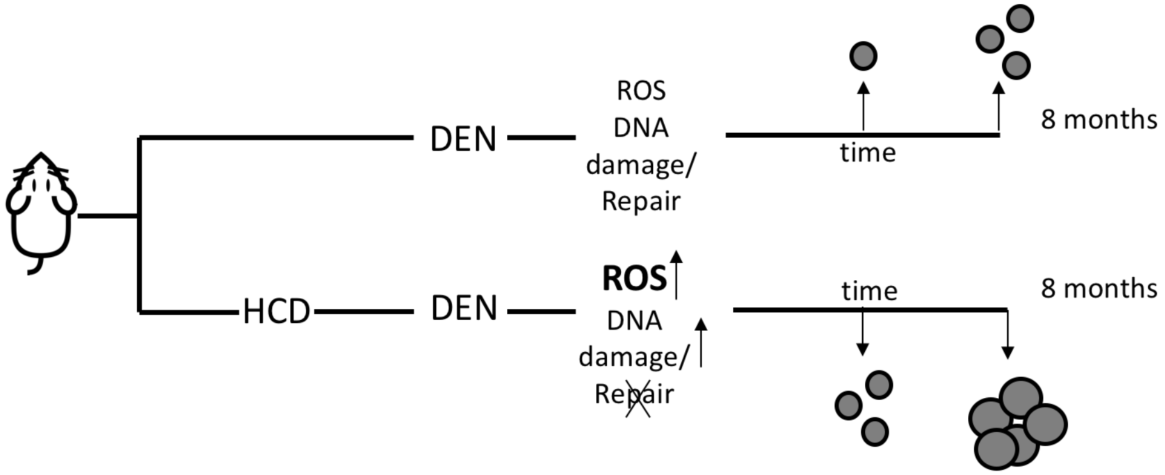

Supplementary Figure 5: Graphical overview of main findings. Cholesterol overload in the liver accelerates the carcinogenic process induced by DEN, increasing ROS content and impairing DNA repair machinery, generating tumors at early times and increased in size comparing with animals under normal diet. HCD, high cholesterol diet; DEN, N-diethylnitrosamine; ROS, reactive oxygen species.

Supplementary Table 1: Antibodies used in the study

Antibody	Company	Catalog number	dilution
Actin	Sigma	A2228	1:1000
Bax	Santa Cruz	6236	1:200
Bcl-2	Santa Cruz	783	1:200
GPX4	Abcam	41787	1:1000
GSTm	Santa Cruz	FL-218	1:200
HO-1	Santa Cruz	5141S	1:200
HSP70	Santa Cruz	1060	1:200
p-ATM (Ser1981)	Cell Signaling	5883	1:1000
p-ATR (Ser428)	Cell Signaling	2853	1:1000
p-BRCA1(Ser1524)	Cell Signaling	9009	1:1000
p-CHK1(Ser345)	Cell Signaling	2348	1:1000
p-CHK2 (Thr68)	Cell Signaling	2197	1:1000
p-ERK1/2 (Thr202/Tyr204)	Cell Signaling	9101	1:1000
p-H2AX (Ser139)	Cell Signaling	9718	1:1000
p-p53 (Ser15)	Cell Signaling	9286	1:1000
gamma-GCS	Santa Cruz	22755	1:200
